# Health system barriers and facilitators influencing the uptake of cervical cancer screening among women in sub-Saharan Africa: systematic review and meta-synthesis

**DOI:** 10.1186/s12913-026-14003-5

**Published:** 2026-01-24

**Authors:** Silas Selorm Daniels-Donkor, Louise Marryat

**Affiliations:** 1 School of Health Sciences, University of Dundee, Scotland, UK; 2 School of Nursing, University of Virginia, Charlottesville, Virginia USA

**Keywords:** Cervical cancer, Cervical cancer screening, Health system, Barriers, Facilitators, Sub-saharan Africa

## Abstract

**Background:**

Cervical cancer ranks among the most frequent cancers and is the fourth leading cause of death among women globally. In Sub-Saharan Africa (SSA), it is the leading cause of cancer-related mortality for women. Although there are primary and secondary preventative interventions available, they are not widely used. This systematic review and meta-synthesis aimed to identify health system barriers and facilitators that influence the uptake of cervical cancer screening among women in SSA.

**Methods:**

A comprehensive search was conducted across four electronic databases to identify English-language papers that highlighted health system barriers and facilitators that impact women in SS. As uptake of cervical cancer screening. Health system barriers and facilitators were extracted and categorized using the World Health Organization’s (WHO) Health Systems Framework: service delivery, health workforce, health information, medical products and technologies, financing, and leadership/governance. The review was carried out following the methodology for systematic reviews and reported using the Preferred Reporting Items for Systematic Reviews and Meta-Analyses (PRISMA) guidelines.

**Results:**

Seven qualitative studies were included in this review. The five categories under which these elements of the health system were categorized were the delivery of health services, the health workforce, health system financing, the health information system, and necessary medications and technology. The most frequent barriers preventing women from getting screened for cervical cancer were those related to the health workforce (lack of appropriate personnel trained in cervical cancer screening), and the health service delivery (lack of screening services in health facilities and long travel distance to screening centres) while the health information system (information from healthcare providers on screening) was identified as a key facilitator for cervical cancer screening.

**Conclusion:**

This review concluded that key health system barriers, such as a lack of properly trained personnel in cervical cancer screening, screening centers, qualified health professionals, and a high cost of consultation, hindered women from accessing screening for cervical cancer in SSA. Resolving these highlighted health system limitations should be the primary focus of strategies to increase cervical screening adoption and usage in SSA. Our review found limited qualitative attention to the treatment and follow-up pathway; strengthening screening programs must include investments in referral systems, diagnostic capacity, and affordable treatment services.

**Supplementary information:**

The online version contains supplementary material available at 10.1186/s12913-026-14003-5.

## Background

Globally, cervical cancer is one of the most common cancers and the fourth leading cause of death among women [1]. Over 570,000 new cases of cervical cancer were diagnosed worldwide in 2018, and 311,000 people died from the disease [2]. Distribution is not even, however, with > 85% of the worldwide burden transpiring in low- and middle-income nations (LMICs) [1]. Prevalence is estimated by age-standardized incidence rate (ASR) to be 23.8 per 100,000 females in low-income areas, compared with 8.3 in developed countries [1, 2]. Africa has the highest regional prevalence and fatality rates: 7–10 times greater than those in Western countries [3, 4].

Cervical cancer screening is available to find precancerous cell abnormalities on the cervix that, if left untreated, could develop into cervical cancer [5]. The WHO initiated a significant campaign to eliminate cervical cancer worldwide in 2018. This elimination approach is based on the three pillars of the Human Papillomavirus (HPV) vaccine, cervical screening, and treatment, with related intervention targets, and is more specifically targeted at reducing cervical cancer. The’90–70-90’ elimination targets state that 90% of women receive adequate treatment for pre-existing conditions, 70% of women obtain a cervical HPV test at least twice in their lifetime, and 90% of adolescent girls receive prophylactic HPV vaccinations [6]. In High-Income Countries (HICs), such as the United Kingdom, primary HPV testing has been highly successful: national implementation in 2019 resulted in an 87% decrease in cytology volumes [7]. The UK,s 70% reduction in cervical cancer death rates since the program’s introduction serves as a proxy for the program’s overall effectiveness [8]. By contrast, prevention programmes in most LMICs have not been widely implemented [9–11].

In LMICs, evidence indicates that as few as two screenings per lifetime may drastically lower the incidence of cervical cancer in populations who do not currently receive screenings [12]. The prevalence of cervical cancer screening in LMICs is only 19% on average, compared with 63% HICs [5]. In SSA, screening is particularly scarce, with evidence indicating that only 10% of women in SSA had access to cervical cancer screening [13]. Cervical cancer was the leading cause of cancer death in the SSA in 2018, accounting for 21.7% of all cancer fatalities among female patients [14].

Despite experiencing a rise in the number of incidents of cervical cancer reported each year in their facilities, most health systems in SSA do not have screening programs for early detection and treatment. Numerous studies demonstrate ongoing challenges to the health system for cervical cancer screening, including a lack of adequately qualified and accessible health professionals, inadequate facilities, and insufficient stocks of medical and healthcare supplies [15–17]. Several systematic reviews have been conducted in SSA to identify *individual-level* barriers and facilitators that influence the uptake of cervical cancer screening among women [5, 18, 19]. These reviews found factors such as educational level, age, contraceptive use, awareness, time, fear of screening procedures, and family support influence the uptake of screening. However, to date, no reviews have explored the impact of the *health system* on cervical screening uptake. This is crucial because removing individual barriers alone is unlikely to encourage the uptake in screening required to reduce cervical cancer prevalence, without corresponding systemic barriers also being addressed. The World Health Organization’s Health Systems Framework identifies six core components of a health system: service delivery, health workforce, health information, medical products and technologies, financing, and leadership/governance. [20]. We adopted this framework to classify the barriers and facilitators identified, as it provides a comprehensive lens to examine how different health system factors influence cervical cancer screening uptake.

## Methods

### Aim

This review aimed to systematically identify health system barriers and facilitators influencing the uptake of cervical cancer screening among women in SSA using the six components of the WHO Health Systems Framework (service delivery, health workforce, health information, medical products and technologies, financing, and leadership/governance).

### Search strategy

A systematic comprehensive search of the literature for qualitative research that explored health system barriers and facilitators influencing the uptake of cervical cancer screening among women in SSA. Qualitative studies were included to capture rich data on health systems’ enablers and impediments to screening as experienced by women or health care professionals. Studies that addressed the opinions and perspectives of health care professionals were included since it was thought that they would have pertinent knowledge of aspects of health systems. The search was conducted in June 2023 using the following electronic databases: CINAHL Plus, Web of Science, and MEDLINE. The ‘OR’ and ‘AND’ operators were used to combine the results of independent subject and text word searches conducted on each database. Supplementary Table 1 presents the search terms used in each of the selected databases. To address potential publication bias, we integrated database searching with backward and forward citation tracking using Google Scholar, and we examined the reference lists of included studies and relevant reviews. Inclusion was restricted to English-language, peer-reviewed studies from January 2013 to June 2023; this limitation is recognized as a potential source of language and publication bias.

### Study selection

Two researchers screened the titles and abstracts independently (SSD-D and LM). Studies were included for further review if they met the inclusion criteria demonstrated in Table 1.

**Table 1:** Inclusion and Exclusion Table for study selection

Inclusion	Exclusion
Qualitative empirical studies	All other studies including quantitative studies, systematic reviews
Focusing on health system barriers and/or facilitators to the utilisation of cervical cancer screening	All other topics, including individual-level factors
Screening of HIV negative women (between the ages of 25 to 64) in SSA	
English language papers	
Full-text article available	
Papers published in peer-reviewed journals between January 2013 and June 2023	

Two researchers (SSD-D and LM) then independently screened papers at the full-text level. Any disagreements were discussed until a conclusion was reached

### Data extraction of study characteristics

One author (SSD-D) extracted the study features onto the data extraction sheet. From every study, the following information was extracted: the authors, the year the work was published, the nation, the sample size, the research techniques, the health system barriers and facilitators, the time the data was collected, and the limits of the qualitative studies. The information extracted from the various studies was cross-checked by another author (LM).

### Quality assessment of studies

Two independent reviewers used the items reflecting conceptual domains from the Critical Appraisal Skills Programme (CASP) quality assessment tool, which were adapted to evaluate the level of quality of the relevant qualitative studies used in this review, as recommended by WHO/Cochrane for qualitative studies [21, 22]. The CASP tool is the most widely used checklist/criteria-based technique to evaluate quality appraisal in synthesizing qualitative data associated with health and social care [22, 23]. The CASP questions’ responses were colour-coded as part of the assessment process to determine their reliability (green: yes, yellow: can’t tell, red: no).

### Data synthesis and analysis

Since the primary goal of this analysis was to identify the health system determinants that affected cervical cancer screening using the six components of the WHO Health Systems Framework, a narrative synthesis approach was used. To determine the aspects of the health system influencing cervical cancer screening among women in SSA, the information retrieved from the included studies was reviewed and re-read. Each health system component identified was categorized according to the six components of the WHO Health Systems Framework.

### Assessment of publication bias and heterogeneity

As a qualitative meta-synthesis, this review rendered typical statistical evaluations for publication bias and heterogeneity inapplicable. We aimed to mitigate publication bias by conducting an extensive search across many databases, employing citation tracking, and reviewing reference lists; the restriction to English-language publications is acknowledged as a limitation. The heterogeneity was analyzed narratively by comparing study settings, participant groups, and screening modalities, while aligning themes with the WHO Health Systems Framework. This methodology highlighted context-specific distinctions in the synthesis.

## Results

### Search outcome

**Fig. 1:**
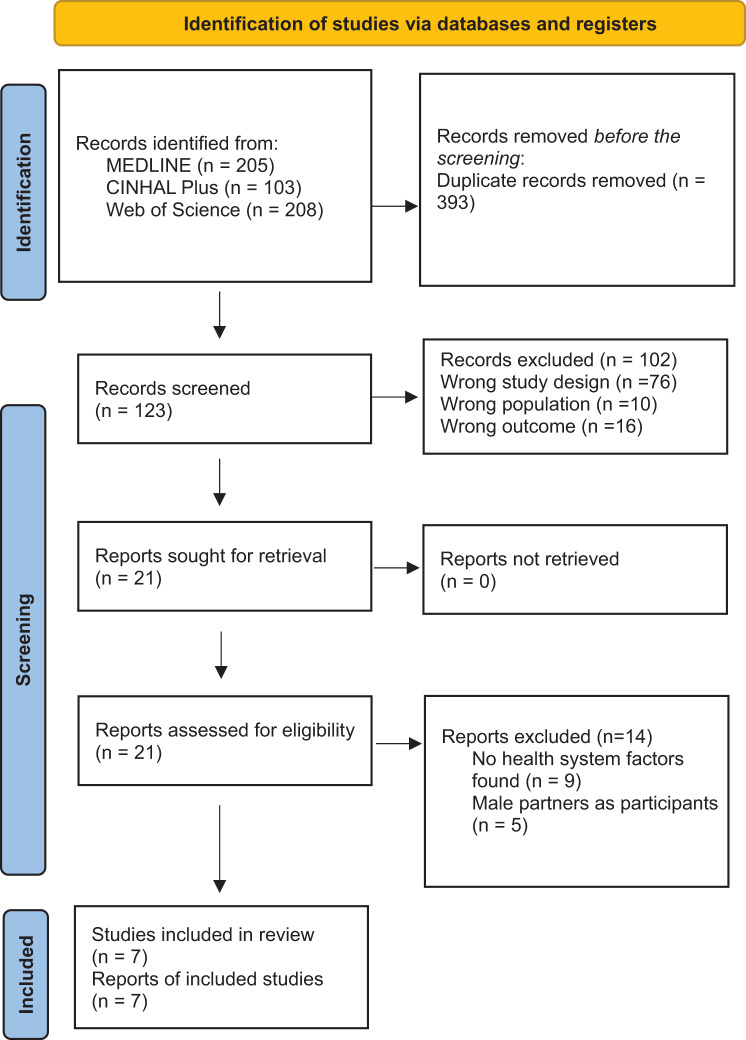
PRISMA 2020 flowchart for finding relevant articles in the database

Five hundred and sixteen articles were identified in the primary database search (MEDLINE (205), Web of Science (208), and CINAHL Plus (103) (Fig. 1)). Duplicates (393) were eliminated. A search in Google Scholar yielded no additional articles to be included in this review. One hundred and twenty-three studies were then reviewed by two researchers at the title and abstract stage, with 102 studies eliminated for failing to meet the inclusion criteria. Twenty-one papers were reviewed at the full-text stage: fourteen studies were removed at this stage because they a) did not specifically address health system elements that were barriers to or facilitators of cervical cancer screening in SSA [9] or included male partners as respondents [5]. Seven papers met the inclusion criteria and were included in the review.

### Quality of studies

The seven papers that made up the review had varied levels of quality. Most of the studies were reliable in reporting the research design, the sampling strategy, the gathering of data, ethical issues, the analysis of the findings, and the goals and relevance of the research. However, most of the studies are very unreliable regarding issues of reflexivity. Table 2 presents an overview of the evaluation.

**Table 2:**
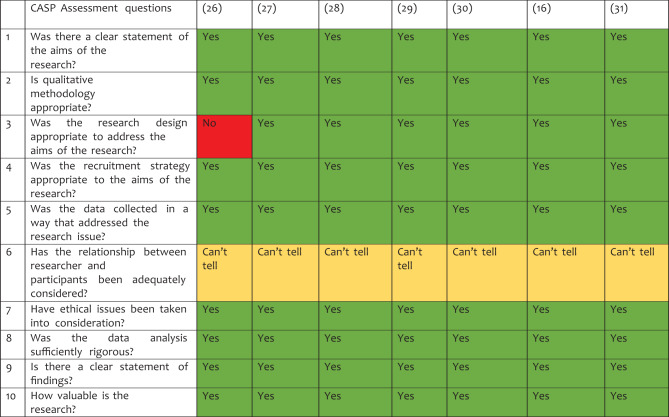
CASP quality assessment for qualitative studies

### Characteristics of the included studies

The seven studies included used semi-structured interviews and focus groups as data collection methods. The included studies were conducted with different research participant demographics: two included women and health care professionals, two women alone, and three included health care professionals alone. The total sample size between the studies was 302 participants, comprising 198 women and 104 healthcare professionals. The studies were conducted in seven SSA countries namely: Tanzania, Rwanda, Ethiopia, Malawi, Kenya, Ghana, and Uganda. Eastern Africa is represented by five studies [24–27], Southern Africa by one [16, 28], and Western Africa by one [29] (Table 3).

**Table 3:** Characteristics and results of included qualitative studies

Authors (Ref)	Country & region*	Study aim	Study design	Sample	Screening modality	Barriers	Facilitators	WHO Building blocks	Limitations
Adewumi et al. [25],	Kenya, Eastern Africa	To assess barriers and facilitators to cervical cancer screening in Western Kenya from the perspectives of community members and healthcare providers.	Exploratory qualitative study	24 community women between 25 to 60 years from communities in Migori County and 12 health providers from 6 districts in Migori.	Pelvic exam	Inadequate screening equipment. The burden on the provider. Lack of personnel trained properly in cervical cancer screening. Male provider performing a screening examination.		Health workforce Essential medicines and supplies.	The study was conducted in Migori County in the Nyanza so the results cannot be generalized to different settings in Kenya.
Binka et al. [29]	Ghana	To explore the barriers to the uptake of cervical cancer screening and treatment in the North Tongu district of Ghana	Exploratory qualitative study	25 women between the ages of 25–65 years living in and around Battor	Pap smear/VIA	Medical professionals make unfavorable sentiments. Diagnostic errors are made by medical experts. Lack of screening and treatment services. High cost of treatment		Health workforce Service delivery Health system financing	Findings cannot be generalized to other settings in the region as it was carried out in only one of the districts.
Gafaranga et al. [26]	Rwanda, Eastern Africa.	To elucidate knowledge about cervical cancer, and identify barriers and motivators or Rwandan women to use cervical cancer screening services.	Descriptive qualitative study	30 women between the ages of 30–59 years. 14 women in Muhima district hospital and 16 women in Nyagasambu health Center.	VIA	Not having health insurance and paying for consultations. Long travel to the screening location. Lack of nearby screening services. Insufficient details regarding the location of screening services. Inadequate testing supplies.	Information obtained from health workers.	Health workforce Essential medicines and supplies. Service delivery Health system financing Health information system.	The participants in this study were women who made it to the health facility and findings cannot be generalized to those who have not visited health facilities.
Lott et al. [24]	Ethiopia, Eastern Africa	To describe cancer control experts’ perspectives regarding the cancer control strategy and implementation of VIA.	Qualitative study	18 health workers in several administrative zones of the Oromia region of Ethiopia: Shewa, West Arsi, Arsi, and Bale	VIA	Lack of screening materials. Lack of infrastructure for screening. Screening centres are understaffed.		Health workforce. Service delivery. Essential medicines and supplies.	The study only included a few knowledgeable participants willing to share their experiences which makes it vulnerable to self-report bias.
Mugassa et al. [16]	Tanzania, Eastern Africa	To examine factors influencing the uptake of cervical cancer screening services among women in Tanzania	Descriptive qualitative study	10 health informants of the reproductive Health-Cancer Unit of MoHCDGEC, the Kinondoni Municipal health System, and the Ocean Road Cancer Institute in the Kinondoni Municipal Council	Pap smear	Shortage of qualified health professionals. Insufficient communication of national information to lesser levels. Instruments and tools are not readily available enough. Inadequate supply of services for cervical cancer screening.		Health workforce Service delivery Essential medicines and supplies. Health information system	The study was conducted in one Municipality so the results cannot be generalized to different settings in Tanzania.
Munthali et al. [28]	Malawi, Southern Africa	To investigate service providers’ perceptions about barriers for women to access cervical cancer screening and early treatment services in Malawi	Qualitative study	53 healthcare providers in 13 districts in Malawi.	Visual Inspection with acetic acid (VIA)	The staffing situation is dire. Insufficient supplies and equipment. Lack of encouragement from the supervisor. Engaging male service providers. Access to medical facilities is far away.		Health workforce Essential medicines and supplies. Service delivery	The researchers did not mention how data saturation was reached
Ndejjo et al. [27]	Uganda, Eastern Africa	To explore community knowledge, facilitators, and barriers to cervical cancer screening among women in rural Uganda.	Exploratory Qualitative study	119 women between 25 49 years in two major rural districts of Bugiri and 11 health workers and administrators from health facilities in the districts.	Not stated	Lack of screening services offered by healthcare providers. Lack of knowledge regarding the availability of screening services. Long travel distance to the screening facilities. Mistreatment by health professionals	Information obtained from health workers. Health facilities offering free screening services.	Service delivery Health workforce Health information system	Findings cannot be generalized to other settings as it was carried out in two major rural districts.

### Barriers

Five (out of six) WHO,s building blocks (health services delivery, health workforce, health system financing, health information systems, and essential medications and technology) were used to classify health system barriers affecting the uptake of cervical cancer screening (Table 4). These will now be discussed in turn.

**Table 4:** Classifying health system *barriers* influencing cervical cancer screening according to the WHO building blocks

WHO Building blocks	Health system barriers influencing uptake of cervical cancer screening	Studies that reported health system barriers
Health Workforce	The burden on the provider. Mistreatment and negative encounters with health professionals. Lack of personnel trained properly in cervical cancer screening. Male provider performing a screening examination. Lack of encouragement from the supervisor. Medical experts make diagnostic errors. Shortage of qualified health professionals.	[25] [27, 29] [16, 25–27] [25, 27–29] [28] [29] [25, 27, 28]
Health service Delivery	Lack of screening services in health facilities. Long travel distance to the screening facilities. Lack of space and infrastructure for screening. Inadequate privacy at screening centres. Unavailability of screening services every day.	[16, 26, 27, 29] [26–29] [24, 28] [29] [28]
Health system Financing	Not having health insurance. Unaffordable consultations and treatment cost. Transportation cost to screening centres.	[26] [26, 27, 29] [27]
Access to Essential Medicines and Technology	Instruments and tools for screening are not readily available enough. Inadequate testing supplies and materials.	[16, 28] [24, 27]
Health information system	Insufficient communication of national information to local levels and failure to utilize the health information system.	[16]
Leadership and Governance		No studies reported findings on this building block.

### Health service delivery

Barriers relating to health service delivery were commonly reported across the studies (reported by 6 studies [16, 24, 26–29]. A major obstacle reported was the absence of screening services in healthcare facilities [16, 26, 27, 29]. In Ghana, for instance, a 46-year-old woman who has never been screened stated:

“I am not aware of any facility that conducts screening for cervical cancer [29].”

Similar to this, a healthcare professional in Uganda concluded that:

“From the mobilizations that we do, some people would like to undergo the screening, but there is no nearby health facility that is offering the service.” [27].

Where facilities did exist, women had to travel long distances to health facilities offering these services [26–29]. For instance, a female respondent said: “Some of us are coming from a rural area where there are no health facilities. We have to come this far before we can come and screen. If only the government can provide more health facilities, it will promote the uptake of cervical cancer screening and treatment in our communities [29]”.

Lack of space and infrastructure for screening [24, 28] and inadequate privacy at screening centers [29] were other factors that hindered women from getting screened for cervical cancer. A respondent, aged 37, who had never undergone screening highlighted that: “If I do not get privacy in the room where the test will be done and if the health workers are not friendly, or if they do not explain things better to me, I might abstain from doing it [29]”.

Another obstacle preventing women from being screened for cervical cancer is the absence of daily screening programs in healthcare institutions. Some service providers expressed the desire that their health centers would offer daily cervical cancer screening and early treatment services, just like supermarkets do [28].

### Health workforce

All seven papers explored barriers relating to the health workforce which were affecting uptake of cervical cancer screening [16, 24–29]. A major impediment to the uptake reported was a lack of appropriately trained staff [16, 24–27]. A healthcare provider in a public hospital in Uganda, for instance, narrated that: “We have two health facilities that offer the service, but these do not have medical officers and the available clinical officers do not have the capacity to screen for cervical cancer thus limiting screening to only one major hospital” [27].

Mistreatment and negative encounters with health professionals, coupled with male health providers doing the screening deterred women from utilizing cervical cancer screening services [25–28]. For example:

“There are doctors who come here once in a while and they call us for screening but they handle you like you are going to give birth. They roughly insert an equipment inside and you feel pain. Even if women come and they hear it’s the same method, very few will go there”. [27]

Previous experience of diagnostic mistakes made by medical professionals were also found to deter women from attending screening [29]. Regarding the incorrect diagnosis, a respondent mentioned: “I have told myself I will never go for any screening programme because one day I went to the hospital with my husband who was then sick and coughing. They ran a test to confirm it was tuberculosis. They advised that I come with all my children to be screened and I was then misdiagnosed with tuberculosis. On another occasion, one of my children was sick and we were all asked to screen, and I was misdiagnosed with sickle cell disease. So, when it comes to screening, I decided not to go. Because, whenever I went, the results were frightening, which were not even true [29].”

### Health system financing

Three studies explored barriers relating to health system financing [26, 27, 29]. Women’s inability to use screening services at healthcare facilities was reported to primarily be caused by the high expense of screening and treatment [26, 27, 29]. This was demonstrated by a respondent: “Some of us cannot pay for the cost. Because they asked us to pay, they end up getting a few people taking part, not everyone can get money to test for it. But if the screening and some of the treatments are for free, a lot of us will get involved. If a patient is put on treatment and recovers, she can become useful to the country. Therefore, the government should regularly help everyone for them to take part in the screening [29].”.

Similar to this, a medical professional in Uganda also claimed that: “I think people who come for cervical cancer screening will need money which they may not have. Although the service is free in government health facilities, sometimes the reagents are not there and so people don’t want to go there. When they come to a private setting, there is a cost attached to the service which can affect utilization” [27].

Women were additionally discouraged from getting screened for cervical cancer due to lack of health insurance and the expense of getting to screening facilities [26, 27]. A participant expressed the following concerning the absence of health insurance: “I had a desire for screening but because of lack of health insurance, I waited until I got it [26]”.

### Essential medications and technology

Five studies contained barriers relating to essential medications and technology [16, 24, 26–28]. The screening centers’ inadequate testing equipment and materials dissuaded women from using cervical cancer screening services [24, 26, 27]

Another barrier preventing women from receiving a cervical cancer screening is the lack of readily accessible tools and instruments [16, 28]. For instance, a 35-year-old Rwandan woman from a remote area reported: “When I reached at the screening service, they told me that the screening tools were not available, and they advised me to come back at another time [26]”.

In a similar vein, a healthcare practitioner in Tanzania highlighted that: “This health facility has a donor dependent budget, therefore the funds for implementing cervical cancer prevention services are not always adequate, making it difficult to acquire adequate tools and instruments required for cervical cancer prevention services [16]”.

### Health information system

Only one study health information system barriers to uptake of cervical cancer screening [16] This study found that failing to use the health information system and inadequate national information dissemination prevented women from accessing screening.

“The health system has not involved much the public or private media sectors in promoting the awareness campaign on cervical cancer prevention, so generally there is low awareness creation on cervical cancer screening services [16]”.

### Facilitators

Evidence around health system facilitators was scarce compared to barriers. Data relating to just two of the WHO,s building blocks was found: health service delivery and health information systems (Table 5).

**Table 5:** Classifying health system *facilitators* influencing uptake of cervical cancer screening according to the WHO building blocks

WHO Building blocks	Health system facilitators influencing the uptake of cervical cancer screening	Studies that reported health system facilitators
Health information system.	Information from healthcare providers	[26, 27]
Health service delivery	Health facilities offering free screening services	[27]
Health Workforce		No studies reported findings on this building block.
Health system Financing		No studies reported findings on this building block.
Access to Essential Medicines and Technology		No studies reported findings on this building block.
Leadership and Governance		No studies reported findings on this building block.

### Health service delivery

One study examined facilitators relating to health service delivery [27]. According to this study, health facilities that provide free screening services to women are more successful in engaging women in screening.

### Health information system

Two studies reported health information systems facilitators [26, 27]. Women were reported to be more likely to access screening for cervical cancer after receiving information from healthcare professionals: “When we come for antenatal care or for health of our children, nurses sensitize us about cervical cancer screening” [26]

## Discussion

This systematic review aimed to summarise the evidence on the health system facilitators and barriers to the utilisation of cervical cancer screening in SSA. This was accomplished by extracting reported health system barriers and facilitators and methodically classifying them per the WHO Health Systems Framework.

The results of this analysis cannot be generalised to the entire SSA population due to the qualitative approach of the studies included in this review [30]. However, the focus group discussions and individual interviews with women and health care professionals resulted in a thorough understanding of the aspects of the health system that, in the participants’ perceptions, either hinder or facilitate the utilisation of cervical cancer screening. Despite the region’s cultural and linguistic diversity, women and health care professionals from various countries in SSA highlighted a number of similar health system hurdles and facilitators to the usage of cervical cancer screening. Barriers were more commonly reported than facilitators in the studies reviewed: five out of 6 of the WHO building blocks were discussed in relation to barriers. The exception to this was the Leadership and Governance building block, which did not have any evidence gathered under it. By contrast, just three studies reported on health system facilitators that impact the uptake of cervical cancer screening; these factors were classified under two of the six WHO building blocks, namely the health information system and health care delivery.

A key barrier highlighted in the review was the Health Workforce. In an effort to eliminate cervical cancer, the WHO has set a global objective of 70% screening coverage [31]. Training professionals in appropriate collection techniques and performing molecular tests for the HPV have been implemented to the standard of cervical cancer screening [32, 33]. Despite this implementation, the current study revealed that low uptake of cervical cancer screening was attributed to a dearth of professionals who were highly qualified for the procedure. This is in line with results from other LMICs [34]. Many LMICs face limitations with their budgets, which hinder the distribution and training of qualified medical personnel needed to assist with cervical cancer screening [35]. The quality of the services provided was also impacted, both directly and indirectly, by the lack of skilled service providers in the institutions: facilities were unable to run daily cervical cancer prevention clinics due to a lack of service providers, resulting in fewer women accessing the services. The dynamic structure of the health system has emphasised how crucial it is to have adequate staff for both the national and lower-level healthcare systems in every country to operate effectively [36].

The absence of cervical screening facilities was a key issue reported concerning health service delivery. This issue has also been raised in HICs, with women in EU Member States highlighting the lack of screening services as a deterrent to screening [37]. Additionally, where services do exist, women can face lengthy journeys to reach such services: this is also a barrier to screening [38]. Other research has found that preventative care for cervical cancer is significantly hampered by the remoteness of rural locations, both in terms of geography and resource availability [39]. It is crucial to expand access to cervical cancer screening programmes within communities since doing so will help to address other barriers within the health system, like the expenses for transport and the difficulty of travelling quite a distance to a facility. Outreach-based models, where screening services are routinely expanded to the community alongside building capacity at smaller health clinics, may assist in this. Availability and accessibility of services alone may not be enough to facilitate screening, however. Thus, additional advertisement through mass media, such as radio and television, should be implemented. For instance, more than 33,000 cervical cancer screening tests were performed in 2008 as a result of widespread media coverage of the disease [40].

Appropriate screening services are also key: for example, when the provider was a woman, the likelihood that women would undergo a cervical cancer screening increased. This is in line with other research e.g. in the EU having a male healthcare provider was a frequently mentioned screening barrier [37] whilst, a Canadian study found that culturally appropriate healthcare services were linked to improvements in cervical cancer screening [41]. This disparity can be addressed by enhancing the competencies of the female health workers, e.g. nurses and midwives.

Out-of-pocket expenses for non-emergency medical treatments, such as cervical screening, are a significant impediment to adoption for women in areas with high levels of poverty [19]. Women’s inability to receive disease screening and treatment may be attributed to poverty [42, 43]. Government subsidies for expenses relating to screening and treatment, as well as extending the availability of screening services to community-level healthcare centres may lower the apparent high cost of screening and treatment and reduce this barrier [34, 44]. By contrast, the provision of free screening services was one of only two facilitators reported.

The one barrier which was not reported in the studies reviewed was ‘Leadership and Governance’, despite this being key to preventing implementation of screening programmes, whether that is through a lack of resource allocation, or through resistance from stakeholders to the implementation of such programmes [45]. Not only a barrier to implementation, however, political will can be a key driver in the success of such screening programmes. This has been seen in the roll out of the HPV vaccine in Rwanda, following lobbying of pharmaceutical companies by the first lady of Rwanda, Jeanette Kagame, leading to the incorporation of the HPV vaccine into the country’s National Immunization Program [45].

Encouragement from healthcare professionals to attend periodic screenings was reported as a key facilitator. Similar findings were found in a review carried out among women in Southeast Asia, which showed that receiving medical professionals’ assistance aided in the adoption of cervical cancer screening [46]. In a Zimbabwean study, nearly half (42.9%) of the participants reported that information from health professionals is the most significant way to convey awareness about cervical cancer and screening programmes [47]. Nurse-midwives in particular, can be essential in boosting the adoption of cervical cancer screening in areas with limited resources because they have effectively led initiatives to promote several comprehensive reproductive health services. Community healthcare providers, an essential group in assisting health systems, particularly in countries with limited resources, can contribute to community mobilisation and enlightenment for cervical cancer procedures [48, 49]. At every health conference, healthcare professionals should be encouraged to inquire about and provide suggestions for opportunistic screening, which may be an effective tactic for educating and motivating women to adopt cervical cancer screening in SSA. Notably, none of the included studies evaluated self-collected HPV sampling. This is an important gap given accumulating evidence that HPV self-sampling increases participation in screening and is acceptable to many women in low-resource settings [50]. Systematic reviews and implementation trials indicate self-sampling can increase reach among under-screened women and mitigate barriers such as the need for pelvic exams, discomfort with male providers, and long travel to facilities, though implementation challenges remain logistics, lab capacity, and follow-up systems [50, 51]. In addition, none of the included qualitative studies described key implementation strategies such as task-shifting, screen-and-treat models, or national screening programs, highlighting an important gap in the evidence base and a priority for future research [52]. Addressing the service delivery and workforce barriers we identified will likely require task-shifting of screening to trained non-physician providers and streamlined screen-and-treat models [52].

Importantly, screening is only the first step: timely diagnosis and treatment are required for population-level impact. The WHO elimination strategy, therefore, includes a target that 90% of women identified with cervical disease receive appropriate treatment [12]. This review found limited qualitative attention to the treatment/follow-up pathway; strengthening screening programs must therefore include investments in referral systems, diagnostic capacity, and affordable treatment services [53]. Health system financing, leadership, and governance as potential facilitators point to the need for adequately funded national screening programs supported by strong political leadership and partnerships, as illustrated by successful HPV prevention initiatives in Rwanda [45].

## Limitations of the study

A significant limitation of this review was the absence of studies on this subject from any of the Central African nations, as well as only one paper from Southern and Western Africa, respectively, resulting in the exclusion of a substantial portion of the target population. We limited inclusion to published qualitative studies, which means that quantitative and mixed-methods evidence on health system determinants of cervical cancer screening uptake was not synthesized in this review. This may have resulted in some data on facilitators and barriers captured in quantitative studies from being missed. Future reviews that integrate qualitative and quantitative evidence could provide additional evidence of system-level determinants. The articles employed in this review were those published in English, while other relevant studies might have been published in other native languages and thus be missing from the review.

## Strengths of the study

The present study is the first systematic review and meta-synthesis to explore health system barriers and facilitators of cervical cancer screening utilisation among women in SSA. The comprehensive search was conducted in numerous databases, used a robust search expression, tracked the publications’ citations, and was reviewed by two researchers.

## Conclusion

The findings of this study inform the caveats at the health system level that impede cervical cancer screening uptake among women in SSA. Priority measures must be taken to improve the health system and get rid of the obstacles preventing cervical cancer screening. Investing in a health workforce of suitable size, comprising employees with the right combination of abilities, who are favored by women, competent, and fairly dispersed to support the provision of cervical cancer screening services, is one of these initiatives. Additionally, a key component of enhancing screening is the accessibility and affordability of suitable, secure, efficient, and high-quality testing supplies, equipment, and other medical items. Moreover, comprehensive integration of cervical cancer screening programs into universal health care is needed. In order to ensure sustainable finance, it is imperative to mobilize domestic resources, enhance the efficiency of the health system, and prevent the impoverished from incurring user fees, thereby protecting their financial security. The study’s results highlight how critical it is to enhance conditions, such as information on screening from healthcare professionals, that encourage SSA women to get screened for cervical cancer. Building strong health information systems that produce accurate data on progress in eliminating cervical cancer can be used for monitoring and evaluation, helping local, national, and international players make better decisions and learn from their experiences.

## Electronic supplementary material

Below is the link to the electronic supplementary material.

Supplementary Material 1

## Data Availability

Not applicable (this manuscript does not report data generation or analysis).

## Abbreviations

ASR: Age-standardized incidence rate
ACS: American Cancer Society
CASP: Critical Appraisal Skills Programme
CINAHL: Cumulative Index to Nursing and Allied Health Literature
LMICs: Low and Middle-Income countries
HICs: High-Income Countries
HIV: Human Immunodeficiency Virus
HPV: Human Papillomavirus
MEDLINE: Medical Literature Analysis and Retrieval System Online
PRISMA: Preferred Reporting Items for Systematic Reviews and Meta-Analyses
SSA: Sub-Saharan Africa
VIA: Visual Inspection with acetic acid
WHO: World Health Organization
