# Supplementary material for: Health system barriers and facilitators influencing the uptake of cervical cancer screening among women in sub-Saharan Africa: systematic review and meta-synthesis
Source: BMC Health Serv Res. 2026 Jan 24;26:265. doi: 10.1186/s12913-026-14003-5 (PMC12911285; doi:10.1186/s12913-026-14003-5)
Supplement: Supplementary file 1 — Supplementary Material 1 [file 12913_2026_14003_MOESM1_ESM.docx]

**Supplementary Table 1: Search terms used in the various selected databases**

| **Database** | **No** | **Search terms (open search)** | **Results** |
| --- | --- | --- | --- |
| **CINAHL Plus** | **1** | TI "vaginal smears” OR papanicolaou OR “pap smear” OR “pap stain” OR “pap test” OR “vaginal smear” OR “mass screening” OR “early diagnosis” OR “cervical cancer screening” OR “cervical cancer examination” OR “early detect*” | 9,502 |
|  | **2** | TI barrie* OR obstacle* OR challeng* OR difficult* OR confront* OR enabler* OR promoter* OR facilitator* OR cost* OR afford* OR transport* OR financ* OR “lack of time” OR inconvenien* OR predictor* OR determinant* OR health system* OR health services accessibilit* or factors or utiliz* or utilis* or uptake | 498,242 |
|  | **3** | TI “sub saharan africa" OR "sub-saharan africa" OR "sub sahara" OR sub-sahara OR SSA OR Angola* OR Benin OR Botswana OR “Burkina Faso” OR Burundi OR Cameroon OR “Cape Verde” OR “Central African Republic” OR Chad OR Comoros OR Congo OR “Congo Democratic Republic” OR “Côte d'Ivoire” OR “Equatorial Guinea” OR Eritrea OR Eswatini or Ethiopia OR Gabon OR “The Gambia” OR Ghana OR Guinea OR “Guinea-Bissau” OR Kenya OR Lesotho OR Liberia OR Madagascar OR Malawi OR Mali OR Mauritania OR Mauritius OR Mozambique OR Namibia OR Niger OR Nigeria OR Rwanda OR “Sao Tome and Principe” OR Senegal OR Seychelles OR “Sierra Leone” OR Somalia OR "South Africa" OR Sudan OR "South Sudan" OR Tanzania OR Togo OR Uganda OR Zambia OR Zimbabwe | 58,157 |
|  | **4** | 1 AND 2 AND 3 | 125 |
|  | **5** | Limited to 2013-2023 | **103** |
| **MEDLINE** | **1** | TI “vaginal smears” OR papanicolaou OR “pap smear” OR “pap stain” OR “pap test” OR “vaginal smear” OR “mass screening” OR “early diagnosis” OR “cervical cancer screening” OR “cervical cancer examination” OR “early detect*” | 38,511 |
|  | **2** | TI barrie* OR obstacle* OR challeng* OR difficult* OR confront* OR enabler* OR promoter* OR facilitator* OR cost* OR afford* OR transport* OR financ* OR “lack of time” OR inconvenien* OR predictor* OR determinant* OR health system* OR health services accessibilit* or factors or utiliz* or utilis* or uptake | 1,817,386 |
|  | **3** | “sub saharan africa" OR "sub-saharan africa" OR "sub sahara" OR sub-sahara OR SSA OR Angola* OR Benin OR Botswana OR “Burkina Faso” OR Burundi OR Cameroon OR “Cape Verde” OR “Central African Republic” OR Chad OR Comoros OR Congo OR “Congo Democratic Republic” OR “Côte d'Ivoire” OR “Equatorial Guinea” OR Eritrea OR Eswatini or Ethiopia OR Gabon OR “The Gambia” OR Ghana OR Guinea OR “Guinea-Bissau” OR Kenya OR Lesotho OR Liberia OR Madagascar OR Malawi OR Mali OR Mauritania OR Mauritius OR Mozambique OR Namibia OR Niger OR Nigeria OR Rwanda OR “Sao Tome and Principe” OR Senegal OR Seychelles OR “Sierra Leone” OR Somalia OR "South Africa" OR Sudan OR "South Sudan" OR Tanzania OR Togo OR Uganda OR Zambia OR Zimbabwe | 247,440 |
|  | **4** | 1 AND 2 AND 3 | 228 |
|  |  | Limited to 2013-2023 | **205** |
| **Web**  **of Science** | **1** | TI “vaginal Smears” OR papanicolaou OR “pap smear” OR “pap stain” OR “pap test” OR “vaginal smear” OR “mass screening” OR “early diagnosis” OR “cervical cancer screening” OR “cervical cancer examination” OR “early detect*” | 40951 |
|  | **2** | TI barrie* OR obstacle* OR challeng* OR difficult* OR confront* OR enabler* OR promoter* OR facilitator* OR cost* OR afford* OR transport* OR financ* OR “lack of time” OR inconvenien* OR predictor* OR determinant* OR health system* OR health services accessibilit* or factors or utiliz* or utilis* or uptake | 17,872,753 |
|  | **3** | “sub saharan africa" OR "sub-saharan africa" OR "sub sahara" OR sub-sahara OR ssa OR Angola* OR Benin OR Botswana OR “Burkina Faso” OR Burundi OR Cameroon OR “Cape Verde” OR “Central African Republic” OR Chad OR Comoros OR Congo OR “Congo Democratic Republic” OR “Côte d'Ivoire” OR “Equatorial Guinea” OR Eritrea OR Eswatini or Ethiopia OR Gabon OR “The Gambia” OR Ghana OR Guinea OR “Guinea-Bissau” OR Kenya OR Lesotho OR Liberia OR Madagascar OR Malawi OR Mali OR Mauritania OR Mauritius OR Mozambique OR Namibia OR Niger OR Nigeria OR Rwanda OR “Sao Tome and Principe” OR Senegal OR Seychelles OR “Sierra Leone” OR Somalia OR "South Africa" OR Sudan OR "South Sudan" OR Tanzania OR Togo OR Uganda OR Zambia OR Zimbabwe | 498,833 |
|  | **4** | 1 AND 2 AND 3 | 2,951 |
|  | **5** | Limited to 2013-2023 | **208** |
